# Stainability of Polished and Glazed Printed Monolithic Zirconia After Coffee Thermocycling

**DOI:** 10.1002/cre2.70246

**Published:** 2025-11-01

**Authors:** Soraya Soleimani, Elaheh Beyabanaki, Kiana Shakeri, Reza Eftekhar Ashtiani, Farhad Tabatabaian

**Affiliations:** ^1^ Department of Prosthodontics, School of Dentistry Hormozgan University of Medical Sciences Bandar Abbas Iran; ^2^ Department of Prosthodontics, School of Dentistry Shahid Beheshti University of Medical Sciences Tehran Iran; ^3^ School of Dentistry Shahid Beheshti University of Medical Sciences Tehran Iran; ^4^ Department of Oral Health Sciences, Faculty of Dentistry University of British Columbia Vancouver Canada

**Keywords:** 3D printing, milling, stainability, thermocycling, zirconia

## Abstract

**Objective:**

This study assessed the impact of the method of manufacturing, surface treatment, and thickness on the stainability of zirconia after thermocycling.

**Methods:**

Twenty 0.5 × 10 mm and 20 1 × 10 mm zirconia disks were fabricated using a milling machine. Also, 20 0.5 × 10 mm and 20 1 × 10 mm zirconia disks were fabricated using a 3D printer. After sintering, samples were subjected to one of the two surface treatments (*n* = 10). Eight study groups were arranged as (1) 0.5 mm printed–polished, (2) 1 mm printed–polished, (3) 0.5 mm printed–glazed, (4) 1 mm printed–glazed, (5) 0.5 mm milled–polished, (6) 1 mm milled–polished, (7) 0.5 mm milled–glazed, and (8) 1 mm milled–glazed. CIELab values were measured with a spectrophotometer before and after 10,000 thermal cycles in coffee solution. ΔE_00_ readings were compared with perceptibility (< 0.8) and acceptability (< 1.8) thresholds. Data were analyzed using three‐way ANOVA and the Games–Howell test (*α* = 0.05).

**Results:**

Although all mean ΔE_00_ values were below the acceptability threshold, only 1 mm printed–glazed zirconia and all milled zirconia samples, except for the 0.5 mm polished group, were below the perceptibility threshold. A significant interaction was found between the production method, surface treatment, and thickness (*p* = 0.002); 0.5 mm printed zirconia, either polished or glazed, and also 1 mm polished printed zirconia exhibited significantly higher ΔE_00_ than their milled counterparts. Moreover, glazing significantly reduced stainability in all zirconia specimens (*p* < 0.05). Also, 1 mm milled–polished zirconia had a lower ΔE_00_ value than its 0.5 mm counterpart (*p* = 0.038).

**Conclusions:**

Printed zirconia had less color stability than milled zirconia. Glazing provided the lower color change for both milled and printed zirconia. Thicker milled–polished zirconia had a better color stability.

**Clinical Significance:**

Although the color stainability of printed zirconia was more than milled zirconia, it showed clinically acceptable color change values along with the milled zirconia, regardless of the method of surface treatment and thickness.

## 1 Introduction

Zirconia provides both strength and esthetic appeal, making it desirable in restorative dentistry. Subtractive manufacturing using computer‐aided design/computer‐aided manufacturing (CAD/CAM) technology is the most common method of manufacturing zirconia restorations in dentistry (Khanlar et al. 2021). However, the milling process generates significant material waste and can cause wear on the milling tools, necessitating frequent replacements. Additionally, creating complex structures through milling can be time‐consuming (Khanlar et al. 2021; Methani et al. 2020). Additive manufacturing, also known as three‐dimensional (3D) printing, has been recently introduced for fabricating dental ceramics (Baysal et al. 2022). This technique potentially provides dental restorations with high accuracy, especially in complex and challenging circumstances, and reduced costs (Baysal et al. 2022; Della Bona et al. 2021; Rekow 2020). Furthermore, the even surfaces and fewer undercuts produced by 3D printing decrease the need for considerable post‐production adjustments (Della Bona et al. 2021; Rekow 2020). Various 3D printing methods have been employed to manufacture zirconia restorations, including vat‐photopolymerization method, including stereolithography (SLA), direct light processing (DLP), selective laser sintering (SLS), selective laser melting (SLM), direct inkjet printing (DIP), nanoparticle jetting, binder jetting, fused deposition modeing (FDM), and direct energy deposition (Alghauli et al. 2024). Several factors, such as solid amount, printing parameters, debinding, and sintering temperature, control layer height and printing speeds during additive manufacturing, and could affect the properties of zirconia, such as the amount of porosity (Branco et al. 2020a; Buj‐Corral et al. 2021). Microscopical studies have shown that the surface of 3D‐printed zirconia has micropores due to the weak bonding between the layers, and also air bubbles trapped in the zirconia paste (Branco et al. 2020b; Lu et al. 2020). However, coating the 3D‐printed zirconia with glaze could lead to the absence of pores and a significant reduction of the surface roughness, much like in milled zirconia (Branco et al. 2020b). Compared to stereolithography, DLP provides faster printing speeds due to its ultrarapid light shifting and integral projection capabilities (Ruggiero et al. 2025). Furthermore, DLP can achieve greater resolution by employing multiple micro‐mirrors, and also cures the entire layer at once with a single projection from the light source (Unkovskiy et al. 2021; Al Hamad et al. 2022). Nevertheless, additively manufactured zirconia restorations have shown acceptable precision, trueness, and clinical performance like milled zirconia.

The esthetic success of zirconia restorations depends on many factors, such as shade, translucency, fluorescence, surface texture, and contour (Tabatabaian et al. 2022; Eftekhar Ashtiani et al. 2023; Thalib et al. 2025; Saker and Özcan 2021; Kontonasaki et al. 2019; Walczak et al. 2019; Rigos et al. 2025; Zhu et al. 2024; Reybod et al. 2025; Manziuc et al. 2019; Alp et al. 2018; Ashy et al. 2021). However, color matching and color stability are often perceived as the most esthetic factors for patient satisfaction. Ensuring the restoration's color stability is crucial for the long‐term success of indirect dental treatments (Alghauli et al. 2024; Eftekhar Ashtiani et al. 2023). The stainability of zirconia is determined by a number of parameters, including ceramic manufacturing method, composition, surface characteristics such as surface roughness and porosity, and staining liquids (Tabatabaian et al. 2022; Rigos et al. 2025; Alp et al. 2018; Ashy et al. 2021). Only one recent study assessed the impact of artificial aging on the color stability of a 3D‐printed polished zirconia ceramic after immersion in staining beverages in comparison to milled zirconia and reported color differences for both materials, with less color change for printed zirconia in comparison to milled zirconia (Rigos et al. 2025).

Considering the introduction of 3D‐printed zirconia in dentistry and the lack of studies on the stainability of this material with different surface treatments and different thicknesses in comparison to milled zirconia, the current study was designed to examine the color change of milled and printed zirconia disks following coffee thermocycling. Three null hypotheses were developed as follows: (1) there is no difference in stainability between milled and printed zirconia after coffee thermocycling, (2) there is no difference in stainability between either printed or milled zirconia with two surface treatments of polishing and glazing after coffee thermocycling, and (3) there is no difference in stainability between two thicknesses of 0.5 mm and 1 mm for either polished or milled zirconia after coffee thermocycling.

## 2 Materials and Methods

**FIGURE 1:**
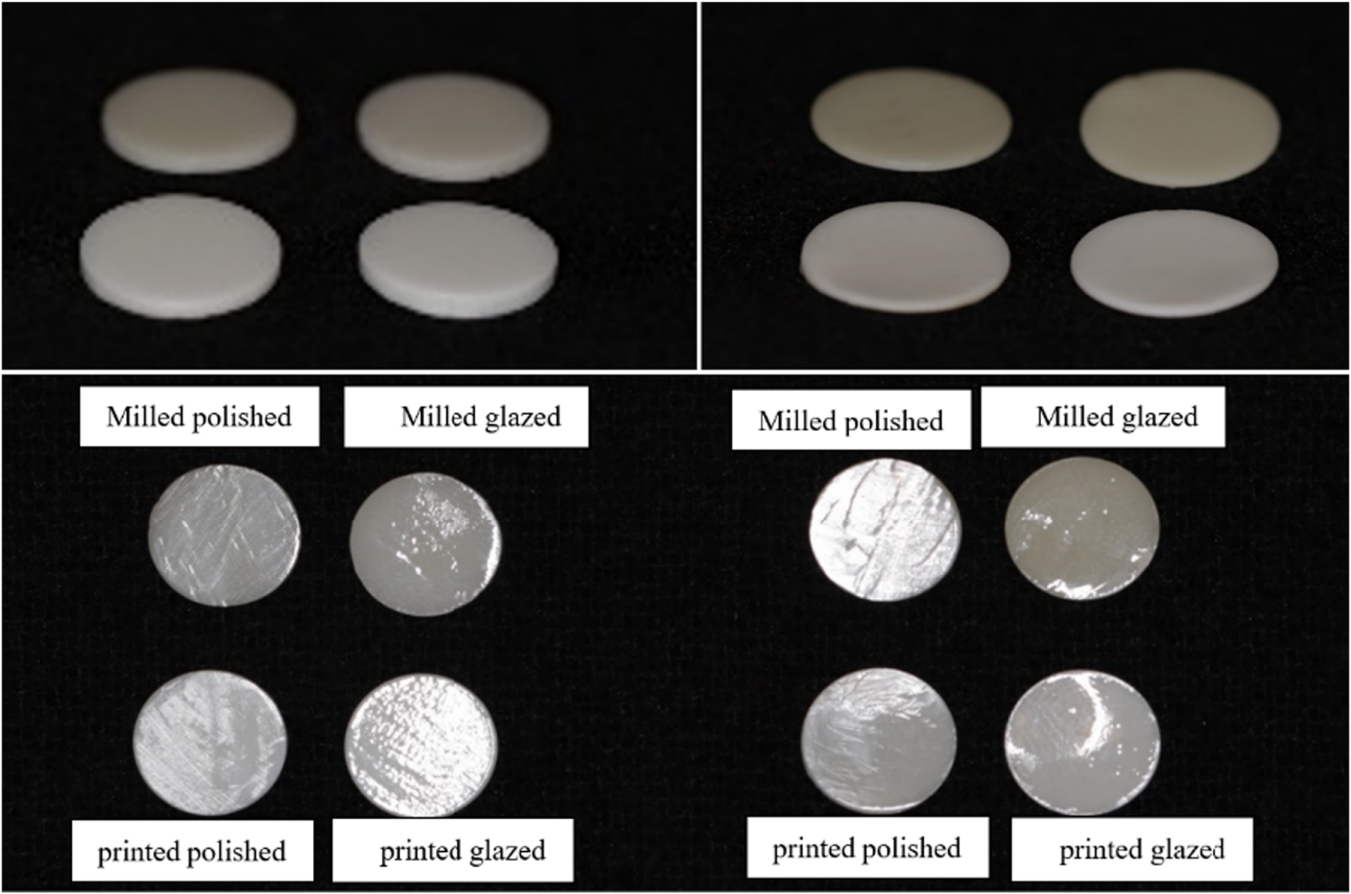
Zirconia disks with different fabrication methods, surface treatments, and thicknesses. Top right: 0.5 mm thickness; top left: 1 mm thickness.

According to a previous study and considering *α* = 0.05, *β* = 0.2, power = 80%, SD = 0.2, *d* = 0.25 (Tabatabaian et al. 2022), sample size was calculated using the formula (*n* = [(Z1 − *α*/2 + Z1 − *β*)2 × 2SD2] ÷ d2). Also, considering *α* = 0.05, *β* = 0.2, power = 80%, SD = 1.63 & 2.24, effect size = 1.4 (Abounassif et al. 2024), sample size was confirmed using G*Power software. Eighty zirconia disks (10 × 1 mm, 10 × 0.5 mm) were considered (*n* = 10 per group) (Figure 1), which were digitally designed using Exocad software (Exocad Dental CAD Galway 3.0), and the standard tessellation language (STL) files were exported for both milling and printing methods.

In the milling group (*n* = 40), a 5‐axis dental milling machine (DWX‐51D, Roland DGA, Irvine, CA) was used to mill the pre‐sintered 3Y‐TZP‐LA monochrome zirconia blocks (Dental Direkt Bio ZX^2^ MedicalZirconia; Germany) based on the STL files. The zirconia chemical composition consisted of >99% wt ZrO_2+_ HfO_2_ + Y_2_O_3_, < 6% wt Y_2_O_3_, < 0.15% wt Al_2_O_3_, and < 1% wt other oxides. After the coloring procedure, the disks were subjected to sintering at a maximum temperature of 1450°C for 9.2 h in a sintering furnace (VITA Vacumat 6000 M; VITA Zahnfabrik GmbH; Germany). The disks were extracted once the furnace temperature dropped below 300°C and allowed to cool down to room temperature. The specimens were then cleaned in an alcoholic solution for 20 min and air‐dried.

In the printing group (*n* = 40), zirconia slurry containing 85% zirconia by volume (3% molyttria–partially stabilized tetragonal zirconia polycrystals) was used. The zirconia slurry bottle was put on the mixer machine (LD‐3D mixer; NextDent; Netherlands). In accordance with the manufacturing instructions, 2 kg of homogenized slurry was poured into the slurry tank of the 3D printer (AON 3D printer; ZIPRO dentistry; South Korea). The slurry was distributed on the building plate using a leveler. The UV light emitted by the projector selectively cured the slurry according to the prescribed STL data. A layer thickness of 30 μm and layer line orientations of 45° were used to enhance the accuracy (Mou et al. 2024). The specimen remained mounted to the building plate for 30 min following the curing of the final layer to guarantee the thorough elimination of the uncured slurry. Once the printing process was finished, the building plate was immersed in isopropyl alcohol for 10 min to remove any uncured slurry that still adhered to the components. The components were separated from the construction disks by removing the supports affixed to their surfaces with tweezers. The final remnant of the slurry was removed from the green body using a soft brush, followed by the spraying of isopropyl alcohol on the surfaces of the disks. Debinding the organic particles was carried out by subjecting the disks to a furnace (Tiger speed, Tiger, South Korea) set at a maximum temperature of 1100°C for an average duration of 14 h. The disks were then colored and sintered for approximately 3 h at 1500°C with a heating–cooling rate of 300°C.

For the coloring process, all specimens were immersed using non‐metal tweezers in an A2 shade liquid (Aqua Coloring Liquid, Zirkonzahn; Italy) for 5 s and subsequently positioned over a piece of cellulose while compressed air was applied to eliminate the excess liquid. The samples were afterward positioned on a firing tray beneath a drying lamp.

Eight study groups (*n* = 10) were arranged as: (1) 0.5 mm milled–polished, (2) 0.5 mm milled–glazed, (3) 1 mm milled–polished, (4) 1 mm milled–glazed, (5) 0.5 mm printed–polished, (6) 0.5 mm printed–glazed, (7) 1 mm printed–polished, and (8) 1 mm printed–glazed. One skilled operator applied surface treatments to the specimens. A two‐step procedure was used to polish both surfaces (Rigos et al. 2025) of the disks using a polishing kit (EVE GmbH; Germany) without water. First, a green cup using light pressure (7.5 min) and then an orange cup using light‐to‐medium pressure, with a single directed motion, was applied to each disk (Baysal et al. 2022). In the glazing group, following the manufacturer's instructions, a thin layer of clear liquid glaze (GC initial glaze; Japan) was applied on both sides of all zirconia disks using a micro‐brush. Each disc was placed on a levelled surface to prevent uneven distribution of liquid material before firing each side of the samples. The disks were fired twice in the ceramic furnace (VITA Vacumat 6000 M; VITA Zahnfabrik GmbH; Germany) at 480°–820°. All samples were cleaned in a bath filled with 98% ethanol for 15 min and then stored at 37°C for 24 h and air dried at room temperature. For ease of tracking and categorization, each sample was assigned a unique number, which was engraved on the back. The accurate thickness of the disks was measured using a digital gauge (293 MDC‐MX Lite; Mitutoyo Corp.). Disks with a thickness outside of 0.5 ± 0.02 mm and 1 ± 0.02 mm were excluded.

Commission Internationale de l'Eclairage (CIElab) values including L* (lightness on *Y*‐axis from 0 to 100), a* (greenness–redness on *X*‐axis from −60 to +60), and b* (blueness–yellowness on Z‐axis from −60 to +60) were measured at baseline before thermocycling with a dental spectrophotometer (Spectro Shade Micro; MHT dental; Germany) by an experienced and blinded operator. The measurements were repeated three times for each specimen on a standard gray background (Tabatabaian et al. 2022). Before each measurement, the device was carefully calibrated based on the following criteria: a light source configured at 2° × 45° (polarized, telecentric, monochromatic), a recording taken at 0° (polarized, telecentric), a measurement area of approximately 18 × 14 mm with a grid of 640 × 480 points, a digital resoltion of 640 × 480, and an optical resolution of approximately 0.03 × 0.03 mm (Tabatabaian et al. 2022). A putty silicone material (Speedex, Coltene, Altstatten, Switzerland) index was customized to the mouthpiece of the spectrophotometer to equalize the conditions of color measurement for all specimens and to prevent the effect of external lights. The substrates and samples were positioned in the center of the putty index over a standard gray card (Tabatabaian et al. 2022). The spectrophotometer was calibrated with its white and green plates, respectively, as recommended by the manufacturer. All color measurements were performed three times at the center of each sample by the same blinded operator, and the average amount was recorded for each specimen.

Subsequently, the specimens were subjected to 10,000 thermal cycles (5°C−55°C) equivalent to 12 months in the coffee solution (Tabatabaian et al. 2022; Rigos et al. 2025). The transfer and dwell times were 10 and 30 s, respectively; 15 ml of coffee (Single Origin Colombia; Starbucks) was added to 177 ml of water to produce the coffee solution as specified by the manufacturer. The solution was refreshed every 12 h. For color evaluation, the specimens were cleaned to remove coffee residue under running water and then dried on cellulose paper. CIELab values were again measured after thermocycling.

The mean of the recorded values was used to calculate ΔE_00_ as follows (Luo et al. 2001):

$$\Delta E_{00}^{*}=\sqrt{{\left(\frac{\Delta L\prime }{k_{L}S_{L}}\right)}^{2}+{\left(\frac{\Delta C\prime }{k_{C}S_{C}}\right)}^{2}+{\left(\frac{\Delta H\prime }{k_{H}S_{H}}\right)}^{2}+R_{T}\frac{\Delta C\prime }{k_{C}S_{C}}\frac{\Delta H\prime }{k_{H}S_{H}}}$$

ΔE_00_ readings were compared to perceptibility (< 0.8) and acceptability (< 1.8) standards for clinical color interpretation (Paravina et al. 2019).

Statistical analysis was conducted using the SPSS software version 26.0 (SPSS Inc., Chicago, IL, USA). The normal distribution of the data was evaluated by the Shapiro–Wilk analysis. Shapiro‐Wilk analysis was used for each group, and it was statistically significant (> 0.05). Considering three variables of method of fabrication (milling vs. printing), thickness (0.5 mm vs. 1 mm), and method of surface treatment (polishing vs. glazing), three‐way ANOVA and Games–Howell were used to compare the mean ΔE_00_ values of eight study groups. To evaluate the color stability of specimens, 1‐sided 1‐sample *t*‐test and software (STATA, StataCorp. LLC, Lakeway, Texas) were used to compare the ΔE_00_ values of different ceramic materials‐thickness‐surface treatment combinations with the perceptibility threshold of ΔE_00_ = 0.8 and the acceptability threshold of ΔE_00_ = 1.8 (*α* = 0.05).

## 3 Results

**FIGURE 2:**
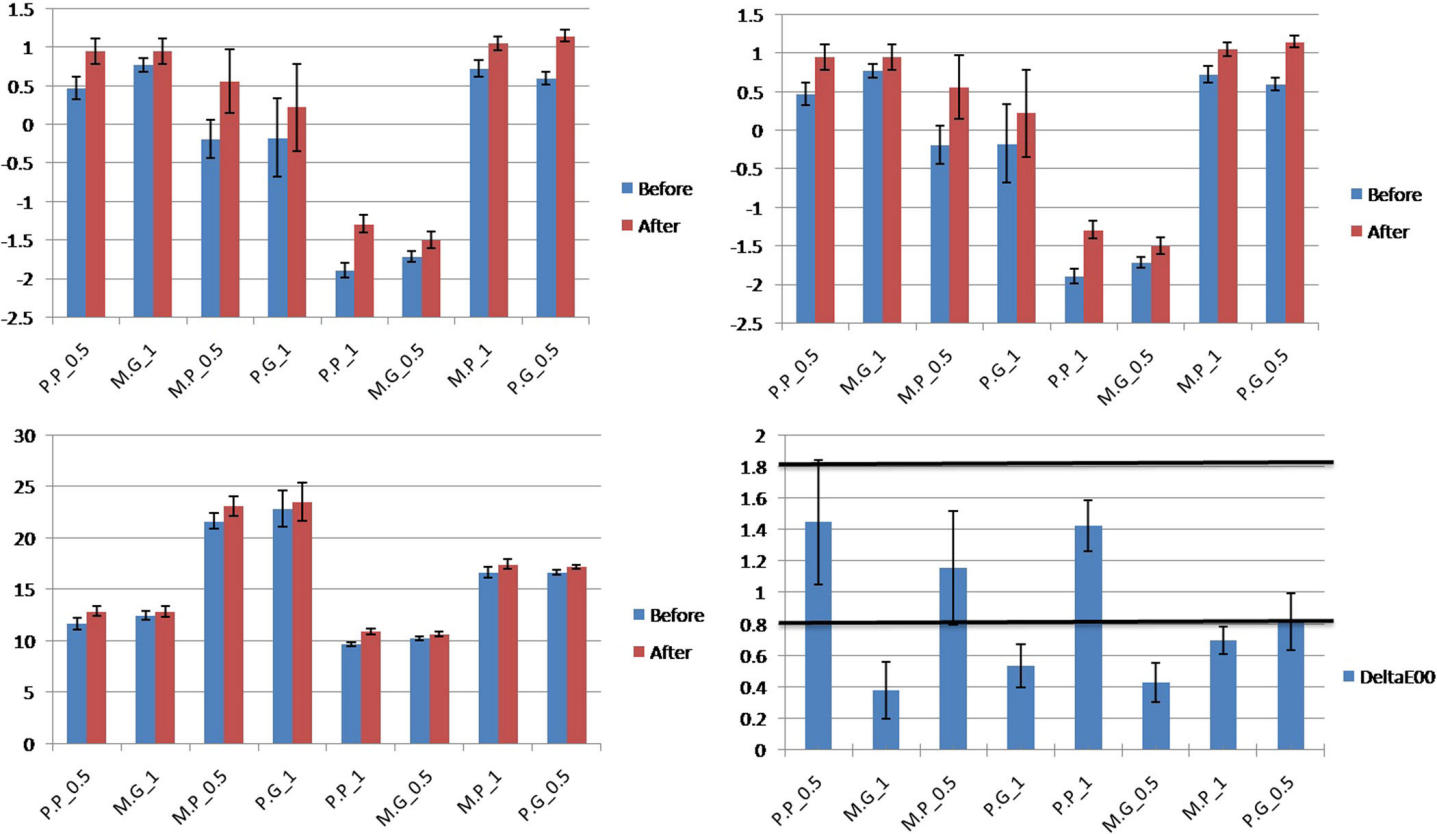
Means values of L* (left upper), a* (right upper), b* (left lower), and ΔE_00_ (right lower) values for study groups before and after coffee thermocycling [P.P; printed–polished, P.G; printed–glazed, M.P; milled–polished, M.G; milled–glazed].

The means CIELab and ΔE_00_ values for the study groups are presented in Figure 2. The production method (milling vs. printing) and surface treatment (polishing vs. glazing) had a significant effect on the color difference. In addition, significant interactions were found between production method and surface treatment, between surface treatment and thickness, and also among production method, surface treatment, and thickness factors (Table 1). The highest and the lowest ΔE_00_ values belonged to the 0.5 mm printed–polished group and 1 mm milled–glazed group, respectively (Figure 2 and Table 2). Moreover, the color change of all zirconia groups was below the acceptability threshold (< 1.8), while 1 mm printed–glazed zirconia and all milled zirconia, except for the 0.5 mm polished group, showed an unperceivable color change (< 0.8).

**Table 1:** Results of three‐way ANOVA on the effects of manufacturing technique, surface treatment, and thickness.

Source	Type III sum of squares	Df	Mean square	*F*	Sig.
Production	0.278	1	0.278	5.319	0.024
Surface	8.253	1	8.253	157.822	0.000
Thickness	0.030	1	0.030	0.577	0.450
Production × Surface	3.058	1	3.058	58.484	0.000
Production × Thickness	0.054	1	0.054	1.036	0.312
Surface × Thickness	0.824	1	0.824	15.749	0.000
Production × Surface × Thickness	0.551	1	0.551	10.536	0.002

**Table 2:** Mean values and SD of DE00 of eight study groups (production × surface treatment × thickness), along with statistical comparisons.

Groups (Number)	Mean (SD)	Significant pair comparison (< 0.05)
Printed.Polished.0.5 (1)	1.4488 (0.39515)	3, 4, 5, 6, 7, 8
Printed.Polished.1 (2)	1.4250 (0.16317)	3, 4, 6, 7, 8
Printed.Glazed.0.5 (3)	0.8156 (0.18099)	7, 8
Printed.Glazed.1 (4)	0.5376 (0.13729)	5
Milled.Polished.0.5 (5)	1.1578 (0.35915)	7, 8
Milled.Polished.1 (6)	0.6980 (0.08546)	7, 8
Milled.Glazed.0.5 (7)	0.4286 (0.12213)	—
Milled.Glazed.1 (8)	0.3784 (0.18098)	—

### 3.1 Effect of Manufacturing Method

According to two‐pair comparison between 1 mm glazed printed and milled zirconia, there was no significant difference (*p* = 0.390). However, 1 mm polished printed zirconia had a higher ΔE_00_ value than 1 mm polished milled zirconia (*p* < 0.001). Also, the results indicated that 0.5 mm printed zirconia had a higher ΔE_00_ value than 0.5 mm milled zirconia in both polished and glazed groups (*p* < 0.05) (Table 3).

**Table 3:** Pairwise comparison of mean ΔE_00_ values between eight study groups.

Group	Group	Mean difference	Std. error	Sig.
Printed.polished.0.5	Printed.polished.1	0.02374	0.13519	1.000
	Printed.glazed.0.5	0.63316	0.13744	0.009
	Printed.glazed.1	0.91118	0.13228	< 0.001
	Milled.polished.0.5	0.29095	0.16886	0.004
	Milled.polished.1	0.75073	0.12785	0.003
	Milled.glazed.0.5	1.02020	0.13079	< 0.001
	Milled.glazed.1	1.07036	0.13744	< 0.001
Printed.polished.1	Printed.glazed.0.5	0.60942	0.07706	< 0.001
	Printed.glazed.1	0.88744	0.06743	< 0.001
	Milled.polished.0.5	0.26721	0.12475	0.440
	Milled.polished.1	0.72699	0.05825	< 0.001
	Milled.glazed.0.5	0.99646	0.06445	< 0.001
	Milled.glazed.1	1.04662	0.07706	< 0.001
Printed.glazed.0.5	Printed.glazed.1	0.27802	0.07184	0.202
	Milled.polished.0.5	0.34221	0.12718	0.207
	Milled.polished.1	0.11757	0.06329	0.598
	Milled.glazed.0.5	0.38704	0.06905	0.001
	Milled.glazed.1	0.43720	0.08094	0.001
Printed.glazed.1	Milled.polished.0.5	0.62023	0.12159	0.005
	Milled.polished.1	0.16045	0.05114	0.094
	Milled.glazed.0.5	0.10902	0.05811	0.582
	Milled.glazed.1	0.15917	0.07183	0.390
Milled.polished.0.5	Milled.polished.1	0.45978	0.11675	0.038
	Milled.glazed.0.5	0.72925	0.11996	0.001
	Milled.glazed.1	0.77941	0.12718	0.001
Milled.polished.1	Milled.glazed.0.5	0.26947	0.04714	0.001
	Milled.glazed.1	0.31963	0.06329	0.004
Milled.glazed.0.5	Milled.glazed.1	0.05016	0.06904	0.500

### 3.2 Effect of Surface Treatment

Analysis of the surface treatment procedures indicated that glazed specimens exhibited lower ΔE_00_ values as compared to polished specimens for both 0.5 mm and 1 mm printed zirconia (*p* < 0.05). Similarly, 0.5 mm and 1 mm glazed milled zirconia had a lower ΔE_00_ value as compared to their polished peers (*p* < 0.05) (Table 3).

### 3.3 Effect of Thickness

The increase in thickness from 0.05 mm to 1 mm resulted in less color stainability merely in polished milled zirconia (*p* = 0.038). However, the effect of thickness on the stainability of printed zirconia was not statistically significant for either polished or glazed specimens (*p* > 0.05) (Table 3).

Moreover, one sample *t*‐test revealed that half of tested combinations had ΔE_00_ values beyond the perceptibility threshold of 0.8 (*p* < 0.05), except for the 1.1−1 mm glazed printed (*p* = 0.999), 1 mm polished milled (*p* = 0.997), 0.5 mm glazed milled (*p* = 1.000), and 1 mm glazed milled (*p* = 1.000) (Figure 2).

## 4 Discussion

Considering the recent introduction of printed zirconia and the lack of comprehensive investigation of its optical properties, this study investigated the effect of manufacturing method, thickness, and surface treatment for DLP‐printed zirconia in comparison to milled zirconia. One recent study evaluated the optical stability of printed zirconia after aging in different beverages such as coffee. However, they only used 2‐mm‐thick zirconia, which is a less common thickness for monolithic zirconia. Considering the clinical application of monolithic zirconia, two thicknesses used in this study were 0.5 mm and 1 mm, which could correspond to the average thickness of laminate veneers and full coverage restorations, respectively (Zhu et al. 2024; Reybod et al. 2025). Moreover, the zirconia used in that study was only polished; however, glazing, as a common process for finishing the surface of monolithic zirconia, was also used in the present study. The first null hypothesis was mostly rejected since there was a significant difference in color stability between milled and printed zirconia samples after coffee thermocycling. The second null hypothesis was completely rejected since there was a significant difference in polished and glazed zirconia stainability after coffee thermocycling. Moreover, the third null hypothesis was partially rejected since there was a significant color difference between the two thicknesses of milled polished zirconia after coffee thermocycling. Considering the acceptability and perceptibility thresholds established by Paravina et al. (2019), most groups had clinically acceptable color changes (0.8 < ΔE_00_ values < 1.8). However, ΔE_00_ values of 1 mm printed–glazed zirconia and all milled zirconia samples except for the 0.5 mm polished group remained undetectable by the eye (< 0.8).

According to the findings about the effect of manufacturing method, 0.5 mm printed zirconia showed less color stability in comparison to 0.5 mm milled zirconia, which could be related to the higher surface porosity of printed zirconia. Several studies have reported that printed surfaces exhibit more porosity and therefore, greater surface roughness as compared to milled surfaces (Branco et al. 2020b; Ruggiero et al. 2025), which might be attributed to the potential evaporation of the solvent in the slurry on the exposed surface during printing before the addition of the next layer (Osman et al. 2017). Motro et al. (2012) claimed an 83% positive and significant correlation between the Ra‐value and ΔE_00_. According to Giugliano et al. (2024), milled zirconia has a higher bulk density and fewer porosities in its structure. Method of manufacturing, grain sizes and porosities (Abounassif et al. 2024), and microstructural composition could affect the optical properties of dental restorations (Manziuc et al. 2019; Alp et al. 2018). The method of milling (wet or dry) might also influence the translucency of milled zirconia (Manziuc et al. 2019). The ceramic particles in the slurry can produce light scattering during vat‐polymerization and lead to unwanted overgrowth of structures that would influence the cure depth and begin unpredictable curing in bilateral directions (Subaşı et al. 2018). This occurrence could ultimately alter the microstructure of zirconia and interrupt its interaction with light. The more porous surface and structure of zirconia produced by additive manufacturing, observed in SEM and XRD analyses, could affect the other optical properties of zirconia in comparison to milled zirconia. Regarding the effect of different surface treatments, there was a less significant color change for glazed zirconia specimens regardless of manufacturing technique and thickness. Glazing, which involves adding a thin layer of low‐fusing porcelain to the surface of ceramic, could reduce the stainability by lowering the surface roughness (Kursoglu et al. 2014). Tabatabaian et al. (2022) also evaluated the effect of the surface treatment method on the color stability of high‐translucent milled zirconia after 10,000 coffee thermal cycles. In line with the findings of this study, they found that ΔE00 values for glazed zirconia were below the perceptibility threshold after coffee thermocycling. However, adjusted and polished zirconia demonstrated clinically acceptable, yet perceptible color changes. Another study reported higher color stability in both pre‐colored and extrinsically colored milled–glazed zirconia; however, there was no significant difference between polished and glazed samples (Giugliano et al. 2024). Similar to monolithic zirconia, glazing increased the color stability of zirconia‐reinforced lithium silicate, lithium disilicate, and also lucite‐reinforced glass ceramics after immersion in coffee solution (Alp et al. 2018; Giugliano et al. 2024; Kursoglu et al. 2014).

Concerning the effect of zirconia thickness, there was a significant difference only between 0.5 mm and 1 mm milled–polished zirconia, and thinner specimens had a greater ΔE_00_ values. Although no other study has evaluated the effect of thickness on the stainability of zirconia with coffee, this finding is in agreement with Thalib et al. (2025) study that reported less color variations and less chromatic shifts in thicker milled zirconia specimens immersed in coloring liquids. However, they used different thicknesses ranging from 1 mm to 2.5 mm. It was revealed that thicker specimens (2.0 mm and 2.5 mm) exhibited superior resistance to color change (lower ΔE values), even following extended immersion and aging time. They also reported that thickness affected color stability more than immersion time. This behavior may be related to Fick's second law of diffusion and the Beer–Lambert law (Thalib et al. 2025; Kontonasaki et al. 2019), which supports the limited penetration depth of coloring liquids and reduced diffusion pathways for ionic migration in thicker zirconia specimens. Thicker zirconia also presents lower translucency due to increased light scattering, which is a positive factor for color masking ability and a negative point for esthetic color matching. Also, it has been demonstrated that depending on the material thickness, different surface finishing and polishing techniques could further influence translucency and color stability of monolithic milled zirconia (Saker and Özcan 2021). Moreover, another study evaluating the color stability of monolithic zirconia, lithium disilicate, and zirconia‐reinforced lithium silicate glass ceramics at thicknesses of 0.5 mm, 0.7 mm, and 1 mm indicated that there was no significant difference among samples with varying thicknesses (Subaşı et al. 2018). However, it is important to note that samples in previous studies underwent fewer thermal cycles compared to the present study (Subaşı et al. 2018; Arif et al. 2019). On the other hand, Alp et al. (2018) revealed that thickness (1 mm, 1.5 mm, and 2 mm) did not affect the color stability of preshaded and externally shaded milled zirconia following 10,000 coffee thermocycling. On the other hand, in a study performed by Manziuc et al. (2019), the color difference observed before and after glazing in milled zirconia with 0.8 mm, 1.5 mm, and 2 mm thicknesses did not follow a consistent pattern. While the 2 mm samples exhibited a smaller color difference compared to the 1.5 mm samples, the 0.8 mm samples displayed the least color difference among all. Additionally, considering the result of studies reporting non‐glazed zirconia samples exhibiting a better color stability as the thickness increased (Thalib et al. 2025; Ashy et al. 2021), it can be assumed that, along with the thickness of samples, the optical interactions of glazing may influence the overall color difference. Based on the findings of this study, it is recommended that milled zirconia with a minimum thickness of 1 mm be used for achieving optimal long‐term color stability. However, there was no significant correlation between specimen thickness and color parameters in printed zirconia, which might reflect the more pronounced influence of the manufacturing method over the thickness. The printed zirconia groups showed a more significant color change in general, and the effect of thickness was less influential for them.

One of the limitations of this study, besides being an in vitro study, which does not completely relate to the oral environment, was the use of one additive method (DLP) for the fabrication of zirconia. Also, only one brand of zirconia, and limited and smaller thicknesses were used. Samples were not cemented with the resin cement, which could also affect the results. In addition, other staining beverages and both pre‐shaded and extrinsically colored zirconia could be used in future studies.

## 5 Conclusion

Within the limitation of this study, the color stability of monolithic zirconia was significantly impacted by the manufacturing methodology (printing vs. milling), surface treatment method (glazing vs. polishing), and thickness (0.5 and 1 mm); 1 mm polished printed zirconia, and also 0.5 mm printed zirconia had more stainability than their milled zirconia peers. Glazed zirconia exhibited less color alterations compared to the polished zirconia. The effect of increasing thickness from 0.5 mm to 1 mm was significant only in milled–polished zirconia (less stainability). However, all specimens exhibited color changes that were below the clinically acceptable threshold (1.8).

## Author Contributions

Soraya Soleimani performed the investigation, collection, and analysis of data. Elaheh Beyabanaki formatted and edited the manuscript and performed the final editing and revision of the manuscript. Kiana Shakeri wrote the original draft. Reza Eftekhar Ashtiani conceptualized and supervised the project. Farhad Tabatabian revised the manuscript.

## Ethics Statement

The authors have nothing to report.

## Conflicts of Interest

The authors declare no conflicts of interest.

## Data Availability

The data that support the findings of this study are available from the corresponding author upon reasonable request.
